# Adenosine modulators and calcium channel blockers as add-on treatment for schizophrenia

**DOI:** 10.1038/s41537-020-00135-y

**Published:** 2021-01-21

**Authors:** Jonne Lintunen, Markku Lähteenvuo, Jari Tiihonen, Antti Tanskanen, Heidi Taipale

**Affiliations:** 1 Department of Forensic Psychiatry, University of Eastern Finland, Niuvanniemi Hospital, Kuopio, Finland; 2 Department of Clinical Neuroscience, Karolinska Institutet, Stockholm, Sweden; 3 Center for Psychiatry Research, Stockholm City Council, Stockholm, Sweden; 4 School of Pharmacy, University of Eastern Finland, Kuopio, Finland

**Keywords:** Schizophrenia, Pharmacology

## Abstract

Relapses remain common among individuals with schizophrenia indicating a need for improved treatments. Creating a completely new drug molecule is expensive and time consuming, and therefore drug repurposing should be considered. Aim of this study was to investigate the risk of psychiatric rehospitalization associated with use of adenosine modulators (AMs) and calcium channel blockers (CCBs) in schizophrenia. Individuals diagnosed with schizophrenia (*N* = 61,889) in inpatient care between 1972–2014 in Finland were included. The follow-up lasted from 1996 to 2017. Main exposures were use of AMs (allopurinol and dipyridamole) and CCBs (dihydropyridines, diltiazem, and verapamil). Thiazide diuretics were used as a negative control. Within-individual models in stratified Cox regression were used and adjusted hazard ratios (HR) with 95% confidence intervals (CIs) are reported. Use of AMs was associated with a reduced risk of psychiatric rehospitalization on drug class level (HR 0.74, 95% CI 0.65–0.84, *P* < 0.0001), as well as on the level of individual drugs (allopurinol HR 0.82, 95% CI 0.70–0.97, *P* = 0.02; dipyridamole HR 0.65, 95% CI 0.55–0.77, *P* < 0.0001). Use of CCBs was associated with a reduced risk of psychiatric rehospitalization on drug class level (HR 0.81, 95% CI 0.77–0.86, *P* < 0.0001). From the different CCBs, only exposure to dihydropyridines was associated with a reduced risk (HR 0.79, 95% CI 0.74–0.84, *P* < 0.0001). No effect was observed for the negative control, thiazide diuretics (HR 0.96, 0.90–1.02, *P* = 0.20). The effects of dipyridamole and dihydropyridines were more pronounced among younger persons and combination of AMs, and CCBs was associated with a lower risk than either drug class as monotherapy. These results indicate a need for randomized controlled trials of these drugs.

## Introduction

Antipsychotics are the basis of schizophrenia treatment^1^. However, despite their use, many patients continue to have symptoms, and the rate of psychiatric rehospitalization among persons with schizophrenia is high^2^. Therefore, improved treatments are needed. Creating a completely new drug molecule is expensive and time consuming, and to overcome these drawbacks drug repurposing should be considered. By drug repurposing, novel targets for drugs that have been used for other indications and proven to be safe can be found^3^. For schizophrenia, adenosine modulators (AMs), such as allopurinol and dipyridamole, and calcium channel blockers (CCBs) have been considered^4^.

Adenosine is a neurotransmitter and neuromodulator that affects both dopaminergic and glutaminergic pathways, both of which are known to be dysfunctional in schizophrenia^1,5^. It is known that adenosine antagonists and agonists produce similar behavioral changes as dopamine agonists and antagonists, respectively^6^. Therefore, there is a theoretical basis for using adenosine agonists, such as allopurinol and dipyridamole, as add-on treatment for schizophrenia. Both of these drugs are believed to increase extracellular adenosine levels^7,8^. Allopurinol is used to treat gout, whereas dipyridamole is an antithrombotic and vasodilator^8,9^. To the best of our knowledge, previous studies on allopurinol and dipyridamole as add-on treatment for schizophrenia have been small randomized controlled trials (RCTs) with somewhat heterogeneous results^6,10–13^. However, a meta-analysis on RCTs found that AMs had beneficial effects as add-on treatment for schizophrenia, when the outcome was measured on the Positive and Negative Syndrome Scale (PANSS) and its subscales^7^.

Calcium is a part of multiple physiological functions, including muscle contraction, heart rate regulation, and neurotransmitter release^14^. Genome-wide association studies have indicated involvement of calcium-related disturbances in the pathogenesis of schizophrenia^15^. Especially polymorphism within *CACNA1C* gene, a gene encoding L-type voltage gated calcium channel subunit, has been described as “one of the most replicable and consistent associations in psychiatric genetics”^16^. Therefore, it is hypothesized that drugs affecting calcium channels could have an effect on treating schizophrenia. Such drugs include CCBs, which are widely used to treat cardiovascular diseases. A recent observational study found that people with severe mental illnesses had a reduced risk of psychiatric hospitalizations and self-harm during CCB use^17^.

The aim of this study was to investigate whether exposure to AMs (allopurinol and dipyridamole) and CCBs (dihydropyridines, verapamil, and diltiazem) was associated with a decreased risk of psychiatric rehospitalization among persons with schizophrenia. We also conducted analyses on nonpsychiatric hospitalizations and all-cause mortality associated with these drugs to assess their safety aspects.

## Results

The characteristics of the study cohort are shown in the Table 1. From the total cohort (*N* = 61,889), 37,775 persons (61%) experienced a psychiatric rehospitalization. The median age was 45 years (IQR 34–57 years) and 50.26% were men. The maximum follow-up time was up to 22 years, with the median being 14.81 years (IQR 7.51–22.00).

**Table 1:** Characteristics of the study population.

	Total cohort (*N* (%))	CCB cohort (*N* (%))	AM cohort (*N* (%))
Number of people in cohort	61,889	10,460 (16.90%)	3410 (5.51%)
Age at baseline, years			
≤35	17,377 (28.08%)	1297 (12.40%)	269 (7.89%)
36–55	27,415 (44.30%)	5631 (53.83%)	1501 (44.02%)
>55	17,097 (27.63%)	3532 (33.77%)	1640 (48.09%)
Median age (IQR), years	45 (34–57)	49 (42–59)	55 (46–65)
Male gender	31,104 (50.26%)	4749 (45.40%)	1679 (49.24%)
Number of psychiatric hospitalizations			
At baseline ≤1	19,002 (30.70%)	3448 (32.96%)	1238 (36.30%)
2–3	18,839 (30.44%)	3051 (29.17%)	949 (27.83%)
>3	24,048 (38.86%)	3961 (37.87%)	1223 (35.87%)
During follow-up ≤1	32,878 (53.12%)	5861 (56. 03%)	2032 (59.59%)
2–3	9926 (16.04%)	1595 (15.25%)	487 (14.28%)
>3	19,085 (30.84%)	3004 (28.72%)	891 (26.13%)
Time since first SZ diagnosis, years			
≤1	23,555 (38.06%)	3168 (30.29%)	1007 (29.53%)
1–5	5579 (9.01%)	812 (7.76%)	245 (7.18%)
>5	32,755 (52.93%)	6480 (61.95%)	2158 (63.28%)
Comorbidities at baseline			
Cardiovascular disease	9651 (15.59%)	2564 (24.51%)	1171 (34.34%)
Diabetes	3208 (5.18%)	875 (8.37%)	376 (11.03%)
Asthma	1733 (2.80%)	342 (3.27%)	136 (3.99%)
Cancer	1766 (2.85%)	328 (3.14%)	169 (4.96%)
Comorbidities during follow-up			
Cardiovascular disease	19,116 (30.89%)	6004 (57.40%)	2496 (73.20%)
Diabetes	8120 (13.12%)	2619 (25.04%)	1054 (30.91%)
Asthma	4856 (7.85%)	1217 (11.63%)	515 (15.10%)
Cancer	6618 (10.69%)	1363 (13.03%)	603 (17.68%)

*CCB* calcium channel blocker, *AM* adenosine modulator, *SZ* schizophrenia.

### Adenosine modulators

**Fig. 1:**
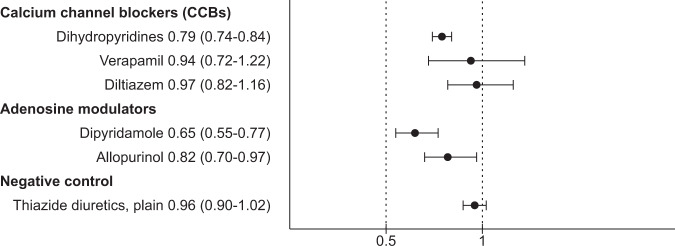
Risk of psychiatric rehospitalization associated with add-on drug use compared with nonuse of add-on drug, within-individual model. Hazard ratios with 95% confidence intervals.

From the total cohort, 3410 (5.51%) persons used AMs (Table 1). Incidence rates of psychiatric hospitalizations are shown in Table 2. AM use was associated with a decreased risk of psychiatric rehospitalization on drug class level (hazard ratios (HR) 0.74, 95% confidence interval (CI) 0.65–0.84, *P* < 0.0001). Of specific drugs, both allopurinol (HR 0.82, 95% CI 0.70–0.97, *P* = 0.02) and dipyridamole (HR 0.65, 95% CI 0.55–0.77, *P* < 0.0001) were associated with a decreased risk of psychiatric rehospitalization (Fig. 1). Superior results were found among the incident cohort (Supplementary Table 3): AM use on drug class level HR 0.47, 95% CI 0.26–0.87, *P* = 0.02 and on the level of individual drugs, allopurinol HR 0.38, 95% CI 0.17–0.87, *P* = 0.02 and dipyridamole HR 0.46, 95% CI 0.22–0.93, *P* = 0.03. When the main analysis was restricted to years 2005–2017 (Supplementary Fig. 1) and the model adjusted for somatic drug use (Supplementary Fig. 2), the results remained similar to the initial analysis. From the two age strata, individuals ≤45 years had a greater decrease of risk associated with AM use than those of >45 years (Supplementary Table 4). Sensitivity analysis with traditional between-individual model was in line with the main analysis (AM HR 0.74, 95% CI 0.68–0.79, *P* < 0.0001; Supplementary Fig. 3).

**Table 2:** Number of users, events, and incidence rates of psychiatric rehospitalizations during add-on drug use.

Add-on drug	Number of users	Use time in person-years	Events	Events/10 person-years (95% CI)
Nonuse of CCBs	61,259	773,313	170,206	2.20 (2.20–2.20)
Any CCB	10,460	50,406	5841	1.16 (1.15–1.17)
Dihydropyridine	9684	45,735	5169	1.13 (1.12–1.14)
Diltiazem	656	2919	453	1.55 (1.51–1.60)
Verapamil	476	1543	188	1.22 (1.16–1.27)
Nonuse of adenosine modulators	61,600	812,112	174,813	2.15 (2.15–2.16)
Any adenosine modulator	3410	11,483	1219	1.06 (1.04–1.08)
Dipyridamole	1899	7263	745	1.03 (1.00–1.05)
Allopurinol	1583	4034	462	1.15 (1.11–1.18)
Nonuse of thiazide diuretics, plain	61,385	782,896	170,510	2.18 (2.17–2.18)
Thiazide diuretics, plain	9026	40,852	5542	1.36 (1.35–1.37)

Individual could contribute to both nonuser and user categories, and different drug substance categories in different time periods during the follow-up.

There were 689 individuals who used more than one CCB and 89 individuals who used both adenosine modulators at the same time.

There were 31 hospitalizations during the time when more than one CCB were used and 12 hospitalizations during the time when both adenosine modulators were used.

*CCB* calcium channel blocker.

**Fig. 2:**
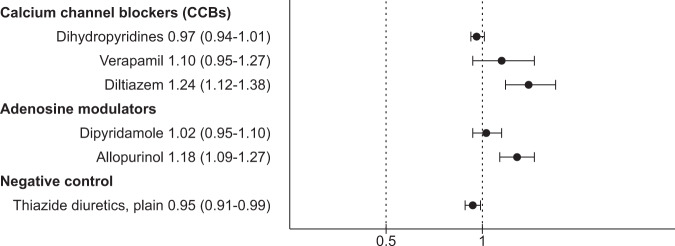
Risk of nonpsychiatric hospitalization associated with add-on drug use compared with nonuse of add-on drug, within-individual model. Hazard ratios with 95% confidence intervals.

**Fig. 3:**
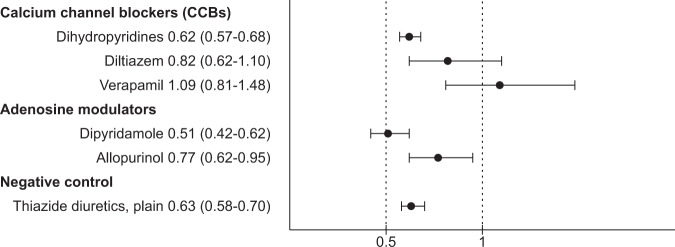
All-cause mortality associated with add-on drug use, adjusted between-individual model. Hazard ratios with 95% confidence intervals.

AM use was associated with an increased risk of nonpsychiatric hospitalization (HR 1.08, 95% CI 1.02–1.15, *P* = 0.01) which was attributable to allopurinol use (HR 1.18, 95% CI 1.09–1.27, *P* < 0.0001), but not for dipyridamole use (HR 1.02, 95% CI 0.95–1.10, *P* = 0.58; Fig. 2). AM use was associated with a decreased risk of all-cause mortality (AM HR 0.61, 95% CI 0.53–0.71, *P* < 0.0001; allopurinol HR 0.77, 95% CI 0.62–0.95, *P* = 0.02; dipyridamole HR 0.51, 95% CI 0.42–0.62, *P* < 0.0001; Fig. 3).

### Calcium channel blockers

From the total cohort, 10,460 persons (16.90%) used CCBs during the follow-up (Table 1). Incidence rates of psychiatric hospitalizations are shown in Table 2. Overall use of CCBs was associated with a reduced risk of psychiatric rehospitalization (HR 0.81, 95% CI 0.77–0.86, *P* < 0.0001). From the different CCB classes, dihydropyridine use was associated with a reduced risk of psychiatric rehospitalization (HR 0.79, 95% CI 0.74–0.84, *P* < 0.0001), but diltiazem (HR 0.97, 95% CI 0.82–1.16, *P* = 0.77) or verapamil (HR 0.94, 95% CI 0.72–1.22, *P* = 0.63) use was not (Fig. 1). Of specific dihydropyridines, use of lercanidipine, amlodipine, and nifedipine were associated with a decreased risk of psychiatric rehospitalization, whereas lack of statistical power limited analysis of other substances (Supplementary Fig. 4 and Supplementary Table 5). Sensitivity analyses among the incident cohort (Supplementary Table 3) had similar results as among the prevalent cohort, and this was also the case for the analyses restricted to years 2005–2017 (Supplementary Fig. 1) and the model adjusted for somatic drug use (Supplementary Fig. 2). From the two age strata, younger strata had a greater decrease in risk than the older strata (Supplementary Table 4). Sensitivity analysis with traditional between-individual model (CCBs HR 0.70, 95% CI 0.68–0.73, *P* < 0.0001; Supplementary Fig. 3) was in line with main analyses.

CCB use was not associated with an increased risk of nonpsychiatric hospitalization (HR 1.00, 95% CI 0.97–1.04, *P* = 0.96; dihydropyridines HR 0.97, 95% CI 0.94–1.01, *P* = 0.17; verapamil HR 1.10, 95% CI 0.95–1.27, *P* = 0.20), with diltiazem being an exception (HR 1.24, 95% CI 1.12–1.38, *P* < 0.0001; Fig. 2). Exposure to CCBs in general (HR 0.66, 95% CI 0.60–0.72, *P* < 0.0001) and dihydropyridines (HR 0.62, 95% CI 0.57–0.68, *P* < 0.0001) was associated with a decreased risk for all-cause mortality, but use of diltiazem (HR 0.82, 95% CI 0.62–1.10, *P* = 0.18) or verapamil (HR 1.09, 95% CI 0.81–1.48, *P* = 0.57) was not (Fig. 3).

Compared with nonuse of both AMs and CCBs, combination therapy was associated with a greater decrease in the risk of psychiatric rehospitalization (HR 0.62, 95% CI 0.48–0.78, *P* < 0.0001) than monotherapy, with AMs (HR 0.70, 95% CI 0.63–0.78, *P* < 0.0001) or CCBs (HR 0.81, 95% CI 0.77–0.86, *P* < 0.0001).

### Thiazide diuretics

Thiazides were used by 9026 persons during the follow-up. Use of thiazides had no association with psychiatric rehospitalization (HR 0.96, 95% CI 0.90–1.02, *P* = 0.20; Fig. 1), but risk of nonpsychiatric hospitalization (HR 0.95, 95% CI 0.91–0.99, *P* = 0.01; Fig. 2) and all-cause mortality (HR 0.63, 95% CI 0.58–0.70, *P* < 0.0001; Fig. 3) were decreased. Traditional between-individual analysis of psychiatric rehospitalization and sensitivity analyses with within-individual model had similar results as the main analysis (Supplementary Figs. 1–3, and Supplementary Tables 3 and 4).

## Discussion

Our results suggest that use of AMs and CCBs as add-on treatment for schizophrenia could be beneficial in decreasing the risk of psychiatric hospitalizations. On the level of individual drugs, the most beneficial results were noted for dipyridamole, and of CCBs, only dihydropyridines were associated with a lower risk. To the best of our knowledge, there are no previous observational studies on this topic on AMs and only one previous study on CCBs as add-on treatment for schizophrenia^17^. Methodologically the previous study was in line with our study as it used rather similar within-individual design, had similar study outcomes and used thiazide diuretics as a negative control. However, the previous study did not separate the different classes of CCBs, but our results on CCBs on drug class level are in line with the results of the previous study, which showed that exposure to CCBs was associated with lower rates of psychiatric hospitalizations. The present study also showed that combination therapy with AMs and CCBs was associated with a greater decrease in the risk of psychiatric rehospitalization compared to monotherapy with the studied drug classes, which suggests that the beneficial effect of these drugs may be exerted through different pathways. It is also noteworthy that both AMs and CCBs were more beneficial among the younger age strata. However, due to fewer events in the combination therapy cohort and younger age strata cohort, these results should be interpreted with caution. Our analyses on risk of nonpsychiatric hospitalization associated with use of add-on drugs showed an increased risk during exposure to AMs, which was attributable to allopurinol, and diltiazem use. This could be, at least to some extent, due to confounding by indication.

Previous studies on AMs as add-on treatment for schizophrenia have been rather small RCTs and the results somewhat differing^6,10–13^. The meta-analysis on using AMs as add-on treatment for schizophrenia included six RCTs (total *N* = 457) with trial durations of 8–12 weeks^7^. AMs were significantly superior to placebo in the PANSS total scores, PANSS positive subscale scores, and in general subscale scores^7^. In terms of specific drugs, allopurinol showed no significant superiority to placebo, while dipyridamole was superior to placebo in PANSS total scores and PANSS general subscale scores^7^. This is in line with our study results where dipyridamole was superior to allopurinol. The mechanism for increasing adenosine levels is different for allopurinol and dipyridamole, which could at least partly explain this difference. In addition, dipyridamole is often used as a combination drug product with acetylsalicylic acid (aspirin), which could also have beneficial effects on schizophrenia^18^.

Possible mechanisms for the association between AMs, and decreased risk of psychiatric rehospitalization may stem from interactions of adenosine with dopamine and glutamate, which both are known to be a part of the etiology and pathophysiology of schizophrenia^1,5,19^. There is increasing evidence suggesting that adenosinergic pathway is a part of the pathology of schizophrenia^5,19,20^. Allopurinol is a xanthine oxidase inhibitor which inhibits purine degradation and consequently, is thought to result in increased adenosine levels^7^. Dipyridamole increases extracellular adenosine levels by inhibiting adenosine reuptake to the erythrocytes, platelets, and endothelial cells^8^. Antipsychotics block dopamine D2-receptors^1^, and since adenosine agonists and antagonists produce opposite effects than dopamine agonists and antagonists, use of adenosine agonists, such as allopurinol and dipyridamole, could be beneficial in schizophrenia^7,11^. The most beneficial effects of AMs were observed among the incident cohort and younger age strata, which suggests that using AMs at the beginning of the illness could be more beneficial. However, due to the sparsity of events and shorter follow-up times in the incident cohort, this result should be interpreted with caution. In addition to adenosine–dopamine and adenosine–glutamate interactions, the beneficial effects of AMs may stem from the inhibition of microglial activation and neuroinflammation. It has been suggested that microglial activation and neuroinflammation are part of the pathogenesis of schizophrenia^21^, and adenosine has modulatory effects on immune and inflammatory systems^22^. Dipyridamole is a known phosphodiesterase inhibitor, possibly possessing anti-inflammatory properties^23–25^. These mechanisms should be considered, especially for dipyridamole, since its penetrance through blood–brain barrier (BBB) could be poor^26^. Allopurinol is thought to be BBB permeable^27^.

Genetic studies have repeatedly shown that alterations affecting *CACNA1C* gene, which encodes the L-type voltage-dependent calcium channel subunit, are associated with schizophrenia^15^. Dihydropyridines, verapamil, and diltiazem target L-type calcium channels^28^ and in this study, we found that exposure to dihydropyridines was associated with a decreased risk of psychiatric rehospitalization. However, exposure to verapamil or diltiazem was not associated with such a decrease. Verapamil and diltiazem have either uncertain or poor passage through BBB, whereas dihydropyridines are known to be permeable through BBB, which may be one possible explanation for this difference^29,30^. Some of the CCBs are substrates and/or inhibitors of permeability glycoprotein (P-gp), which is an important factor on BBB permeability^31^. P-gp is an efflux transporter that limits the entry of foreign substances, including various antipsychotics, into the central nervous system (CNS) and thus, prevents the CNS effects of these drugs^31,32^. Therefore, concomitant use of P-gp inhibiting CCBs with antipsychotics could increase the antipsychotic permeability to the brain^31,32^. However, exposure to verapamil, a known inhibitor of P-gp, was not associated with a decreased risk of psychiatric rehospitalization. One possible explanation for this inconsistency could be related to the statistical power of our study; use of verapamil and diltiazem was scarce compared to use of dihydropyridines (Table 2). Statistical power could also explain differences observed among different dihydropyridines (Supplementary Fig. 4), since number of users varied between different dihydropyridines: over 95% of CCB users used amlodipine, felodipine, lercanidipine, or nifedipine. (Supplementary Table 5). Taken together, our results on CCBs as a drug class are in line with the results of the similar recent observational study conducted in Swedish population^17^.

The strengths of this observational study include a large nationwide cohort of tens of thousands of patients with schizophrenia and the maximum follow-up time was over 20 years. Since our study population included all persons with schizophrenia treated in inpatient care between 1972 and 2014 in Finland, there is no selection bias for cohort inclusion, except for a small group of patients treated only in outpatient care. Thus, our results are generalizable to high-income countries, which provide medications for free or with very low copayment for patients with schizophrenia. Drug use was modeled with PRE2DUP method^33^, which has been shown to produce highly reliable estimates of drug use^34^. Our primary analyses utilized a within-individual model in which all time-invariant factors, such as genetics are controlled for in the design and by that way the common sources of bias in observational studies are minimized. We also conducted several sensitivity analyses with the within-individual model, such as additional adjustments to control for the passage of time (divided age strata, temporal order of treatments, time since cohort entry) and additional somatic medication use, and performed traditional adjusted between-individual analysis for the main outcome (psychiatric rehospitalization). Minor differences between within-individual and between-individual analyses are most likely due to confounding from the factors that are not recorded in the nationwide registers and thus, cannot be adjusted for in between-individual models.

One limitation of our study is that due to lack of time periods when persons were using AMs or CCBs, but not antipsychotics, we could not compare if AMs or CCBs had been effective without antipsychotics. Other limitations of our study are related to the nature of data which is stored in the nationwide registers. Register-based data lack information on many clinically important factors, such as indication of drug use and severity of the symptoms during specific drug exposures and thus, residual confounding may exist. Since it is plausible that patients may use their medications when they are doing well, it is crucially important to use a negative control to address this issue. Our results showed that thiazide diuretics, drugs used mostly for cardiovascular diseases, but without any previously reported psychotropic effects, did not have any effect on risk of psychiatric rehospitalization. This indicates that the beneficial effects associated with dipyridamole and dihydropyridines are not due to chance. As our study is of observational nature, it cannot speak to causal effects. Thus, also the discussion on possible drug mechanisms is only of theoretical nature.

Schizophrenia remains to be a complex psychiatric disease, which causes individual suffering, disability, and high costs to society. Persons with schizophrenia have an increased incidence of multiple somatic diseases and shorter life expectancy^35^, and drugs that have beneficial effects on both physical and mental health could significantly improve treatment outcomes. AMs and CCBs have been used for decades and their pharmacokinetics, pharmacodynamics, and adverse effects are well known. Drug repurposing is a cost-effective and safe method for developing pharmacotherapies, and our results suggest that especially dipyridamole and dihydropyridines could have a beneficial effect in relapse prevention, and their efficacy should be studied in RCTs.

## Methods

### Study population

All Finnish residents have a personal identification number which allows linkage between several registers. Study cohort was identified from the Hospital Discharge register as treated due to schizophrenia (ICD-10 codes F20, F25, ICD-8, and -9 295*) in inpatient care in Finland during 1972–2014. The Hospital Discharge register includes all inpatient hospital stays in Finland. The prevalent cohort included 61,889 persons and the incident cohort 8342 persons^36^. Incident cohort was identified as persons with the first diagnoses of schizophrenia during 1996–2014 and who had not used antipsychotics in the preceding year before diagnoses. For the prevalent cohort the follow-up started on January 1, 1996 and for the incident cohort at the first discharge from inpatient care. The follow-up ended at death or on December 31, 2017 whichever occurred first.

### Exposure

The main exposures were use of AMs and CCBs. AMs included allopurinol and dipyridamole. Febuxostat, a drug belonging to AMs, was excluded from the study due to minimal number of users in our dataset. CCBs included dihydropyridines, diltiazem, and verapamil. We used plain thiazide diuretics as a negative control, since there is no evidence suggesting that thiazide diuretics have any effects on treating schizophrenia. Drug products that had thiazide diuretic combined with another drug were used for model adjustment. Details are in Supplementary Table 1.

Information of drug use was derived from the Prescription Register, which includes reimbursed drug dispensations. Drug use modeling method PRE2DUP is based on the calculation of a sliding average of daily dose in defined daily doses, and it takes into account dose changes, regularity of drug dispensations, possible stockpiling of drugs, and hospital stays^33^. When the method considers whether a drug use period is continuing or not (whether there is a break in use), it uses expert-defined parameters, which have been assigned for each drug package individually, and upper and lower limits for dose defined by clinical recommendations. PRE2DUP method has been validated by comparing PRE2DUP results with interview-based information on drug use^34^. For negative control (plain thiazides) analyses, drug products that had thiazide diuretic combined with another drug were used for model adjustment.

### Outcomes

Main outcome was psychiatric hospitalization (ICD-10 codes F20–29), which indicates relapse in schizophrenia. Secondary outcomes were nonpsychiatric hospitalization (ICD-10 codes other than F00-F99) and all-cause mortality, which were analyzed to observe possible unfavorable effects on physical health.

### Covariates

In within-individual model, individuals act as their own control and, therefore, time-invariant covariates are automatically controlled for in the study design. Main within-individual models were adjusted for the following time-varying covariates: use of antipsychotics (ATC code N05A excluding lithium), mood stabilizers (carbamazepine [N03AF01], valproic acid [N03AG01], lamotrigine [N03AX09), and lithium [N05AN01]), benzodiazepines and related drugs, so called Z-drugs (N05BA, N05CD, and N05CF), and antidepressants (N06A), temporal order of treatments and time since cohort entry. Sensitivity analyses were conducted for the main outcome to adjust for potential time-dependent variations in use of somatic medication, which may impact on the risk of outcomes. These somatic medications were continuously updated in the models and included of the following drugs: metformin (A10BA02) and other oral anti-diabetics (A10B), statins (C10AA), betablockers (C07), angiotensin-converting enzyme inhibitors and angiotensin II receptor blockers (C09), and nonsteroidal anti-inflammatory drugs (NSAIDs M01A excluding glucosamine M01AX05).

Between-individual models were adjusted for gender, age at the cohort entry, time since the first schizophrenia diagnosis, the number of previous psychiatric hospitalizations, comorbidities, and drug use as listed in Supplementary Table 2.

### Statistical analyses

Hospitalization-based outcomes were analyzed in within-individual model, where each individual acted as their own control as exposure periods were compared within the same person. Follow-up time was reset to zero after each outcome event to allow comparison of treatment periods within each individual. By comparing the risk of outcome during different exposure periods for the same individual, instead of between individuals, this approach minimizes selection bias, which is introduced by unmeasured prognostic differences between treatment groups. Persons who had variation in exposure and who had an outcome event during the follow-up contributed to the within-individual analyses. Within-individual models were analyzed with stratified Cox regression^37^. Mortality and other between-individual comparisons were analyzed with traditional Cox regression and comparisons were made between subjects (all individuals contributed into analyses).

In within-individual analyses, HR were calculated for the hospitalization-based outcomes when the person was using an add-on drug compared to the time periods when the add-on drug was not used, in three different exposure categories, (1) AMs, (2) CCBs, and (3) thiazides. We conducted several sensitivity analyses for the main outcome (within-individual models): (1) an analysis with the incident cohort to control for survival bias, (2) an analysis restricting the follow-up to most recent follow-up years 2005–2017 to control for temporal factors, such as changes in overall treatment practices holistically, and (3) an analysis stratified into two age strata (age ≤45 and age >45 at cohort entry) to control for the possible age-related and survival differences. Dihydropyridines were also analyzed on the level of individual CCBs by comparing their use to nonuse of any CCBs. A traditional between-individual analysis for the main outcome was also performed, which was adjusted time dependently for a wide variety of somatic medications to further minimize confounding by somatic conditions. Combination use of AMs and CCBs was also conducted by comparing each drug category in “monotherapy” and in combination use to the nonuse of both categories.

The project was approved by the Ethics Committee of the Finnish National Institute of Health and Welfare (dated 4 December 2013; 8/2013). Further permissions for this research project were granted by pertinent institutional authorities at the Finnish National Institute for Health and Welfare (permission THL/1466/6.02.00/2013), the Social Insurance Institution of Finland (34/522/2013), and Statistics Finland (TK53-305-13). According to Finnish legislation, informed consent is not required for register-based studies using pseudonymized data.

### Reporting summary

Further information on research design is available in the Nature Research Reporting Summary linked to this article.

## Supplementary information

Supplementary material

REPORTING SUMMARY

## Data Availability

The datasets analyzed in this study are not publicly available due to participant privacy and security concerns. Researchers can apply access for these data from the register holders: the Social Insurance Institution of Finland (Prescription Register), the Finnish National Institute for Health and Welfare (Hospital Discharge Register) and Statistics Finland (Causes of Death Register).

## Code availability

The codes that support the findings of this study are available from the corresponding author upon a reasonable request.
