# Supplementary material for: Adenosine modulators and calcium channel blockers as add-on treatment for schizophrenia
Source: NPJ Schizophr. 2021 Jan 21;7:1. doi: 10.1038/s41537-020-00135-y (PMC7820462; doi:10.1038/s41537-020-00135-y)
Supplement: Supplementary file 1 — Supplementary material [file 41537_2020_135_MOESM1_ESM.pdf]

Supplementary Content

Lintunen J, Lähteenvuo M, Tiihonen J, Tanskanen A, Taipale H. Adenosine Modulators and Calcium Channel Blockers as Add-On Treatment for Schizophrenia.

Supplementary Figure 1. Risk of psychiatric re-hospitalization associated with add-on drug use compared with non-use of add-on drug when the follow-up was restricted to years 2005-2017, within-individual model. Hazard ratios with 95% confidence intervals.

Supplementary Figure 2. Risk of psychiatric re-hospitalization associated with add-on drug use compared with non-use of add-on drug, within-individual model. Hazard ratios with 95% confidence intervals. Additionally and time-dependently adjusted for somatic drug use (blood glucose lowering drugs, excluding insulin (A10B), anti-inflammatory and antirheumatic products, non-steroids (M01A, excluding M01AX05 (glucosamine)), HMG CoA reductase inhibitors (C10AA), beta-blockers (C07), angiotensin-converting enzyme (ACE) inhibitors and angiotensin II receptor (ATII) blockers (C09)).

Supplementary Figure 3. Risk of psychiatric re-hospitalization associated with add-on drug use compared with non-use of add-on drug, adjusted between-individual model. Hazard ratios with 95% confidence intervals.

Supplementary Figure 4. Risk of psychiatric re-hospitalization associated with use of different dihydropyridines compared with non-use of CCBs, within-individual model. Hazard ratios with 95% confidence intervals.

Supplementary Table 1. Different thiazides, CCBs, adenosine modulators and psychotropics in the study.

Supplementary Table 2. Covariates used for adjusting the models.

Supplementary Table 3. Risk of psychiatric re-hospitalization associated with add-on drug use compared with non-use of add-on drug, within-individual model among the incident cohort. Hazard ratios with 95% confidence intervals and P-values. Number of users, events and incidence rates of psychiatric re-hospitalizations during add-on drug use are also shown.

Supplementary Table 4. Risk of psychiatric re-hospitalization associated with add-on drug use compared with non-use of add-on drug in two age strata, among those aged ≤45 years and those aged >45 years at cohort entry, within-individual model. Hazard ratios with 95% confidence intervals, P-values, number of users and events of psychiatric hospitalizations.

Supplementary Table 5. Number of users, events and incidence rates of psychiatric re-hospitalizations during specific dihydropyridine use. Non-use of calcium channel blockers (CCBs) as reference.

Supplementary Figure 1. Risk of psychiatric re-hospitalization associated with add-on drug use compared with non-use of add-on drug when the follow-up was restricted to years 2005-2017, within-individual model. Hazard ratios with 95% confidence intervals.

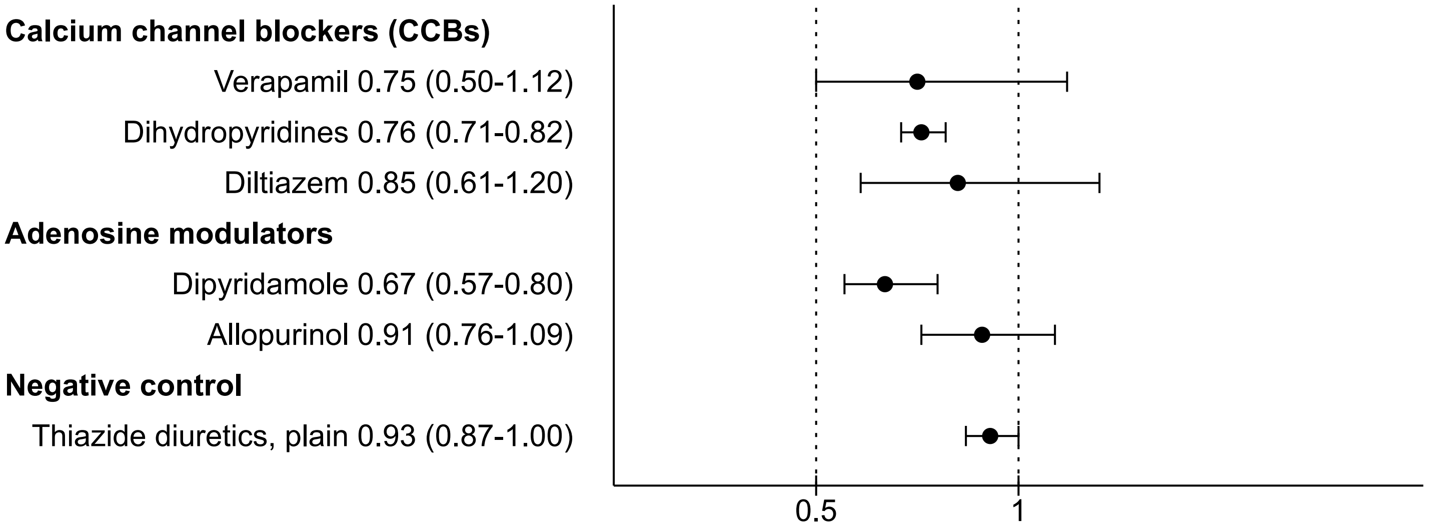

Supplementary Figure 2. Risk of psychiatric re-hospitalization associated with add-on drug use compared with non-use of add-on drug, within-individual model. Hazard ratios with 95% confidence intervals. Additionally and time-dependently adjusted for somatic drug use (blood glucose lowering drugs, excluding insulin (A10B), anti-inflammatory and antirheumatic products, non-steroids (M01A, excluding M01AX05 (glucosamine)), HMG CoA reductase inhibitors (C10AA), beta-blockers (C07), angiotensin-converting enzyme (ACE) inhibitors and angiotensin II receptor (ATII) blockers (C09)).

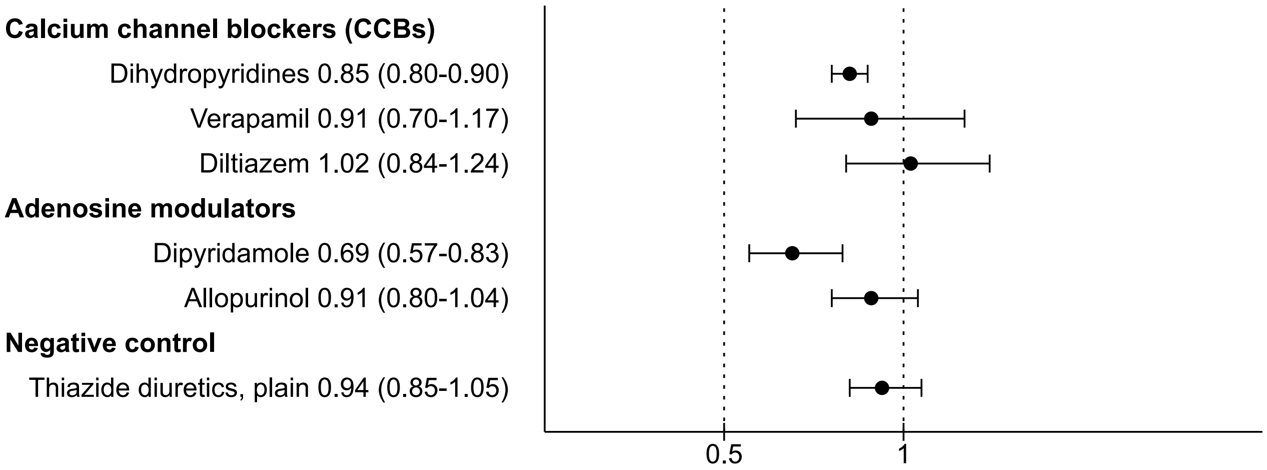

Supplementary Figure 3. Risk of psychiatric re-hospitalization associated with add-on drug use compared with non-use of add-on drug, adjusted between-individual model. Hazard ratios with 95% confidence intervals.

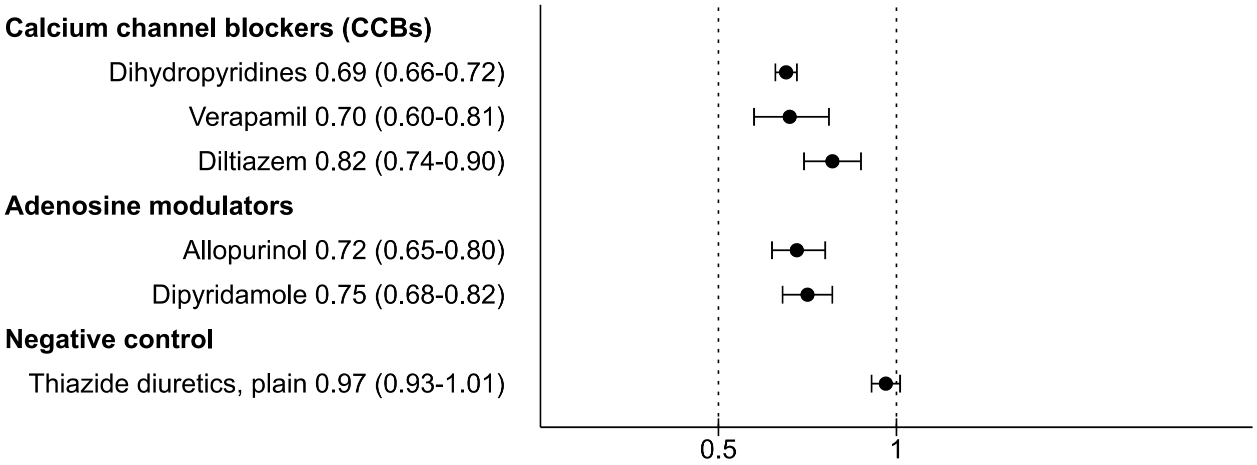

Supplementary Figure 4. Risk of psychiatric re-hospitalization associated with use of different dihydropyridines compared with non-use of calcium channel blockers, within-individual model. Hazard ratios with 95% confidence intervals.

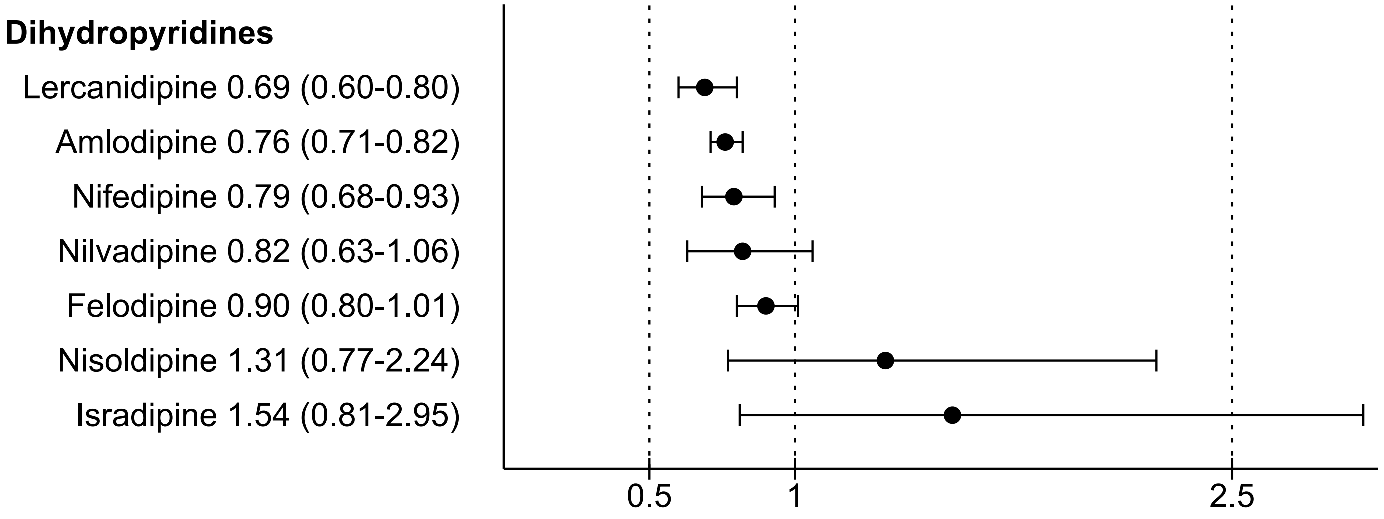

Supplementary Table 1. Different thiazides, calcium channel blockers, adenosine modulators and psychotropics in the study.

Drug type	Drug	ATC
Thiazide diuretics, plain	Cyclothiazide	C03AA09
	Hydrochlorothiazide	C03AA03
Thiazide diuretics, combinations	Bisoprolol and thiazides	C07BB07
	Candesartan and diuretics	C09DA06
	Enalapril and diuretics	C09BA02
	Eprosartan and diuretics	C09DA02
	Hydrochlorothiazide and potassium-sparing agents	C03EA01
	Lisinopril and diuretics	C09BA03
	Losartan and diuretics	C09DA01
	Metoprolol and thiazides	C07BB02
	Nebivolol and thiazides	C07BB12
	Olmesartan medoxomil and diuretics	C09DA08
	Quinapril and diuretics	C09BA06
	Ramipril and diuretics	C09BA05
	Telmisartan and diuretics	C09DA07
	Trichlormethiazide and potassium-sparing agents	C03EA02
	Valsartan and diuretics	C09DA03
Calcium channel blockers, dihydropyridines	Aliskiren and amlodipine	C09XA53
	Aliskiren, amlodipine and hydrochlorothiazide	C09XA54
	Amlodipine	C08CA01
	Amlodipine and diuretics	C08GA02
	Atenolol and nifedipine	C07FB03
	Atorvastatin and amlodipine	C10BX03
	Atorvastatin, amlodipine and perindopril	C10BX11
	Candesartan and amlodipine	C09DB07
	Enalapril and lercanidipine	C09BB02
	Felodipine	C08CA02
	Isradipine	C08CA03
	Lercanidipine	C08CA13
	Lisinopril and amlodipine	C09BB03
	Metoprolol and felodipine	C07FB02
	Nifedipine	C08CA05
	Nilvadipine	C08CA10
	Nimodipine	C08CA06
	Nisoldipine	C08CA07
	Olmesartan medoxomil and amlodipine	C09DB02
	Olmesartan medoxomil, amlodipine and	C09DX03
	Perindopril and amlodipine	C09BB04
	Perindopril, amlodipine and indapamide	C09BX01
	Ramipril and amlodipine	C09BB07

	Ramipril and felodipine	C09BB05
	Rosuvastatin and amlodipine	C10BX09
	Telmisartan and amlodipine	C09DB04
	Valsartan and amlodipine	C09DB01
	Valsartan, amlodipine and hydrochlorothiazide	C09DX01
Calcium channel blockers, diltiazem	Diltiazem	C08DB01
Calcium channel blockers, verapamil	Trandolapril and verapamil	C09BB10
	Verapamil	C08DA01
	Verapamil, combinations	C08DA51
Adenosine modulators, dipyridamole	Dipyridamole	B01AC07
	Dipyridamole, combinations	B01AC30*
Adenosine modulators, allopurinol	Allopurinol	M04AA01
Antidepressants	Non-selective monoamine reuptake inhibitors	N06A
	Selective serotonin reuptake inhibitors	
	Monoamine oxidase inhibitors, non-selective	
	Monoamine oxidase A inhibitors	
	Other antidepressants	
Antipsychotics	Phenothiazines with aliphatic side-chain	N05A
	Phenothiazines with piperazine structure	
	Phenothiazines with piperidine structure	
	Butyrophenone derivatives	
	Indole derivatives	
	Thioxanthene derivatives	
	Diphenylbutylpiperidine derivatives	
	Diazepines, oxazepines, thiazepines and oxepines	
	Benzamides	
	Other antipsychotics	
Mood stabilizers	Carboxamide derivatives	N03AF
	Fatty acid derivatives	N03AG
	Lithium	N05AN01
	Other antiepileptics	N03AX
Anxiolytics	Benzodiazepine derivatives	N05BA
Hypnotics and sedatives	Benzodiazepine derivatives	N05CD
	Benzodiazepine related drugs	N05CF

Abbreviation: ATC = Anatomical Therapeutic Chemical classification system.
* In this dataset, all B01AC30 combinations were dipyridamole + acetylsalicylic acid (ASA).

Supplementary Table 2. Covariates used for adjusting the models.

Covariate	Definition	Model
Temporal order of treatments	Order of treatment continuously updated in the models, categorized as no treatment, 1st, 2nd, 3rd, >3rd	WT, BT
Concomitant use of psychotropic drugs	Antidepressants (N06A), antipsychotics (N05A excluding lithium), benzodiazepines and related drugs (N05BA, N05CD, N05CF) and mood stabilizers (valproate, carbamazepine, lamotrigine, lithium) continuously updated in the models	WT, BT
Other drug use	Opioid analgesics (N02A), non-opioid analgesics (N02BE01, M01A excluding M01AX05 (glucosamine)), lipid lowering drugs (C10A, C10B), anticholinergic anti-Parkinson drugs (N04AA) and prior use of long-acting injection antipsychotic (long-acting injection formulations extracted)	BT
Comorbidities	Cardiovascular disease (I00-I99), diabetes (E10-E14, or antidiabetic use ATC-code A10), asthma / COPD (J42-44), cancer (C01-C99), substance abuse (F10-F19, K86.0, K70 or ATC-codes N07BB, N07BC01, N07BC02, N07BC51) and suicidality (X60-X84, Y10-Y34)	BT
Age	≤ 35, 36-55, > 55 years	BT
Gender	Male or female	BT
Time since first schizophrenia diagnosis	≤ 1, 1-5, > 5 years	BT
Time since cohort entry (=time since 1.1.1996 for prevalent and since the first diagnoses for incident cases)	In years, categorized as ≤ 1, > 1-5, > 5, continuously updated in the models	WT, BT
The number of previous psychiatric hospitalizations time-dependently	≤ 1, 2-3, 4-5, 6-9, ≥10	BT
**Covariates specific for certain sensitivity analyses**		
Oral anti-diabetics<br>Non-steroidal anti-inflammatory drugs (NSAIDs)<br>Statins<br>Beta-blockers<br>ACE-inhibitors and ATII-blockers	A10B<br>M01A, excluding glucosamine M01AX05<br><br>C10AA<br>C07<br>C09	Within-individual model additionally adjusted for a range of somatic medications
Age strata	Analyses conducted separately for those aged ≤ 45 years and for those aged > 45 years at cohort entry	Age-stratified within-individual model

Abbreviations: WT = within-individual model, BT = between-individual model

Supplementary Table 3. Risk of psychiatric re-hospitalization associated with add-on drug use compared with non-use of add-on drug, within-individual model among the incident cohort. Hazard ratios with 95% confidence intervals and P-values. Number of users, events and incidence rates of psychiatric re-hospitalizations during add-on drug use are also shown.

**Add-on drug**	**Use compared with no use, HR (95% CI) and P-value**	**Number of users**	**Use time in person-years**	**Events**	**Events / 10 person-years (95% CI)**
Non-use of CCBs	Reference	8 279	85 199	20 756	2.44 (2.43-2.45)
Any CCB	0.77 (0.62-0.97), P = 0.02	1 031	3 739	464	1.24 (1.21-1.28)
Dihydropyridines	0.75 (0.59-0.94), P = 0.01	973	3 553	441	1.24 (1.20-1.28)
Diltiazem	1.35 (0.23-7.75), P = 0.74	37	91	6	0.66 (0.49-0.83)
Verapamil	2.37 (0.77-7.37), P = 0.13	37	84	17	2.03 (1.72-2.33)
Non-use of adenosine modulators	Reference	8 315	87 999	21 126	2.40 (2.39-2.41)
Any adenosine modulator	0.47 (0.26-0.87), P = 0.02	320	914	89	0.97 (0.91-1.04)
Allopurinol	0.38 (0.17-0.87), P = 0.02	162	398	16	0.40 (0.34-0.46)
Dipyridamole	0.46 (0.22-0.93), P = 0.03	163	486	69	1.42 (1.31-1.53)
Non-use of thiazide diuretics, plain	Reference	8 297	86 165	20 890	2.42 (2.41-2.43)
Thiazide diuretics, plain	0.98 (0.77-1.25), P = 0.86	731	2 778	332	1.20 (1.15-1.24)

Abbreviation: CCB = calcium channel blocker

Individual could contribute to both non-user and user categories, and different drug substance categories in different time periods during the follow-up.

There were 47 individuals who used more than one CCB and 11 individuals who used both adenosine modulators at the same time.

There were no psychiatric hospitalizations during the time when more than one CCB were used and 4 hospitalizations during the time when both adenosine modulators were used.

Supplementary Table 4. Risk of psychiatric re-hospitalization associated with add-on drug use compared with non-use of add-on drug in two age strata, among those aged ≤45 years and those aged >45 years at cohort entry, within-individual model. Hazard ratios with 95% confidence intervals, P-values, number of users and events of psychiatric hospitalizations.

Add-on drug	Age ≤45 at cohort entry date	Age >45 at cohort entry date
Dihydropyridines	HR (95% CI): 0.72 (0.65-0.79)<br>P < 0.0001<br>No. of users: 3 724<br>Events: 2 415	HR (95% CI): 0.90 (0.83-0.98)<br>P = 0.02<br>No. of users: 5 960<br>Events: 2 754
Diltiazem	HR (95% CI): 0.66 (0.48-0.91)<br>P = 0.01<br>No. of users: 92<br>Events: 114	HR (95% CI): 1.20 (0.97-1.48)<br>P = 0.10<br>No. of users: 564<br>Events: 339
Verapamil	HR (95% CI): 0.63 (0.41-0.97)<br>P = 0.04<br>No. of users: 121<br>Events: 52	HR (95% CI): 1.25 (0.89-1.75)<br>P = 0.20<br>No. of users: 355<br>Events: 136
Allopurinol	HR (95% CI): 0.68 (0.54-0.86)<br>P = 0.001<br>No. of users: 531<br>Events: 208	HR (95% CI): 1.09 (0.86-1.38)<br>P = 0.47<br>No. of users: 1 052<br>Events: 254
Dipyridamole	HR (95% CI): 0.65 (0.48-0.88)<br>P = 0.005<br>No. of users: 327<br>Events: 213	HR (95% CI): 0.76 (0.63-0.92)<br>P = 0.006<br>No. of users: 1 572<br>Events: 532
Thiazide diuretics, plain	HR (95% CI): 0.94 (0.85-1.03)<br>P = 0.16<br>No. of users: 3 087<br>Events: 2 529	HR (95% CI): 1.05 (0.97-1.14)<br>P = 0.26<br>No. of users: 5 939<br>Events: 3 013

Supplementary Table 5. Number of users, events and incidence rates of psychiatric re-hospitalizations during specific dihydropyridine use.

**Add-on drug**	**Number of users**	**Use time in person-years**	**Events**	**Events / 10 person-years (95% CI)**
Non-use of CCBs	61 259	773 313	170 206	2.20 (2.20-2.21)
Amlodipine	6 822	25 989	2 932	1.13 (1.12-1.14)
Felodipine	1 582	8 938	1 070	1.20 (1.17-1.22)
Lercanidipine	1 420	5 172	500	0.97 (0.94-0.99)
Nifedipine	894	3 530	436	1.24 (1.20-1.27)
Nilvadipine	252	1 299	142	1.09 (1.04-1.15)
Nisoldipine	105	323	59	1.83 (1.68-1.98)
Isradipine	78	456	25	0.55 (0.48-0.62)
Nimodipine	2	0.30	0	0
